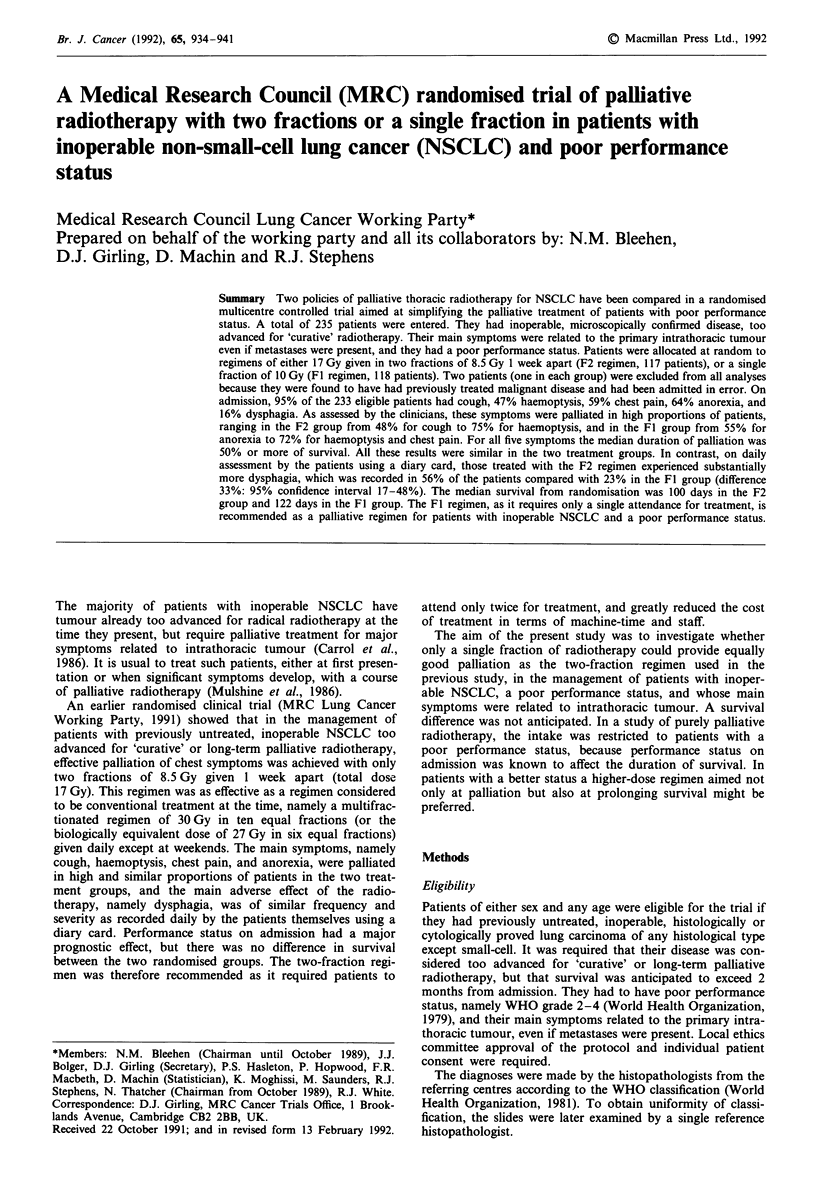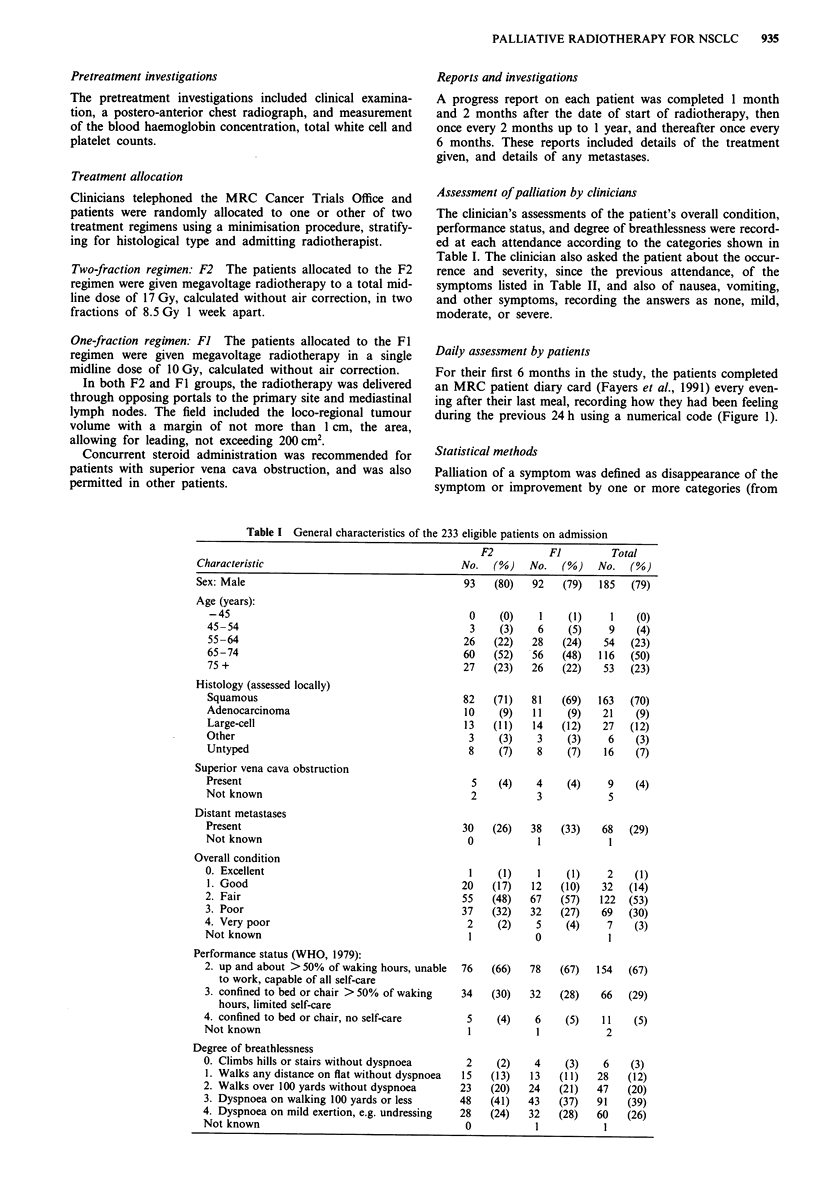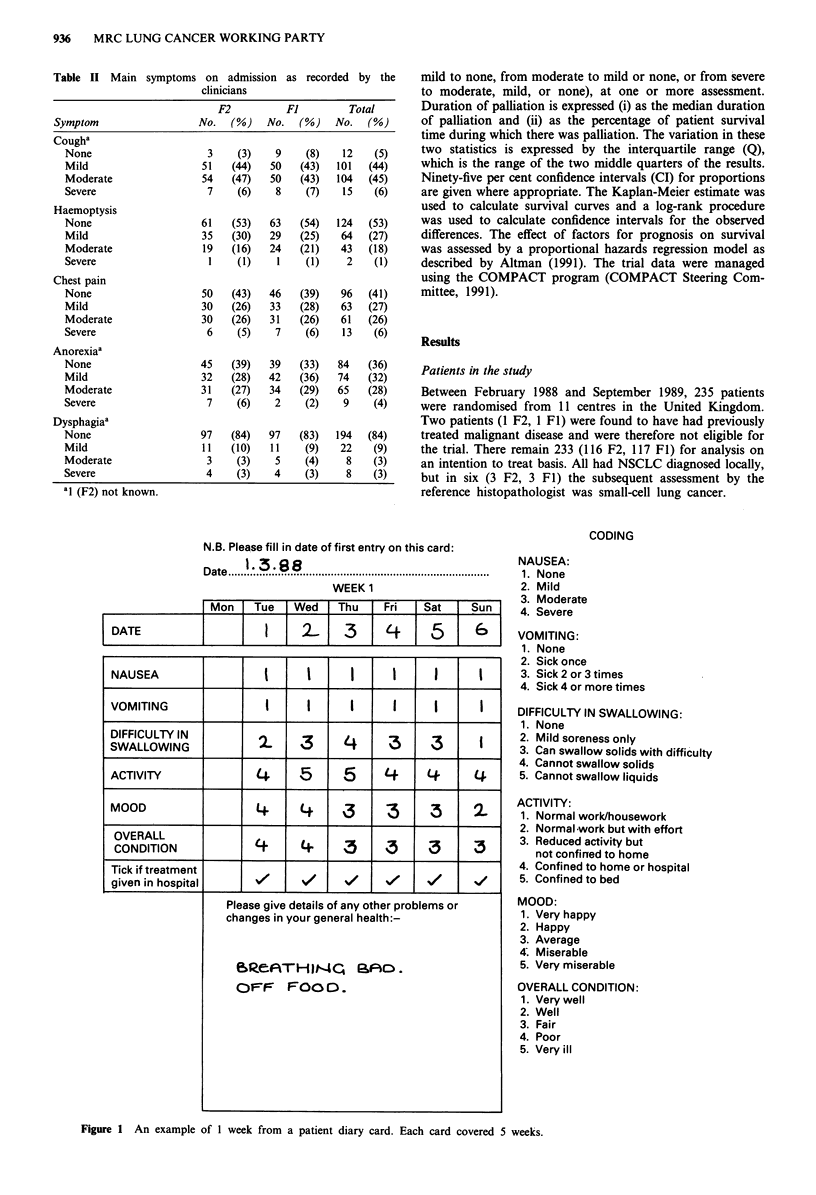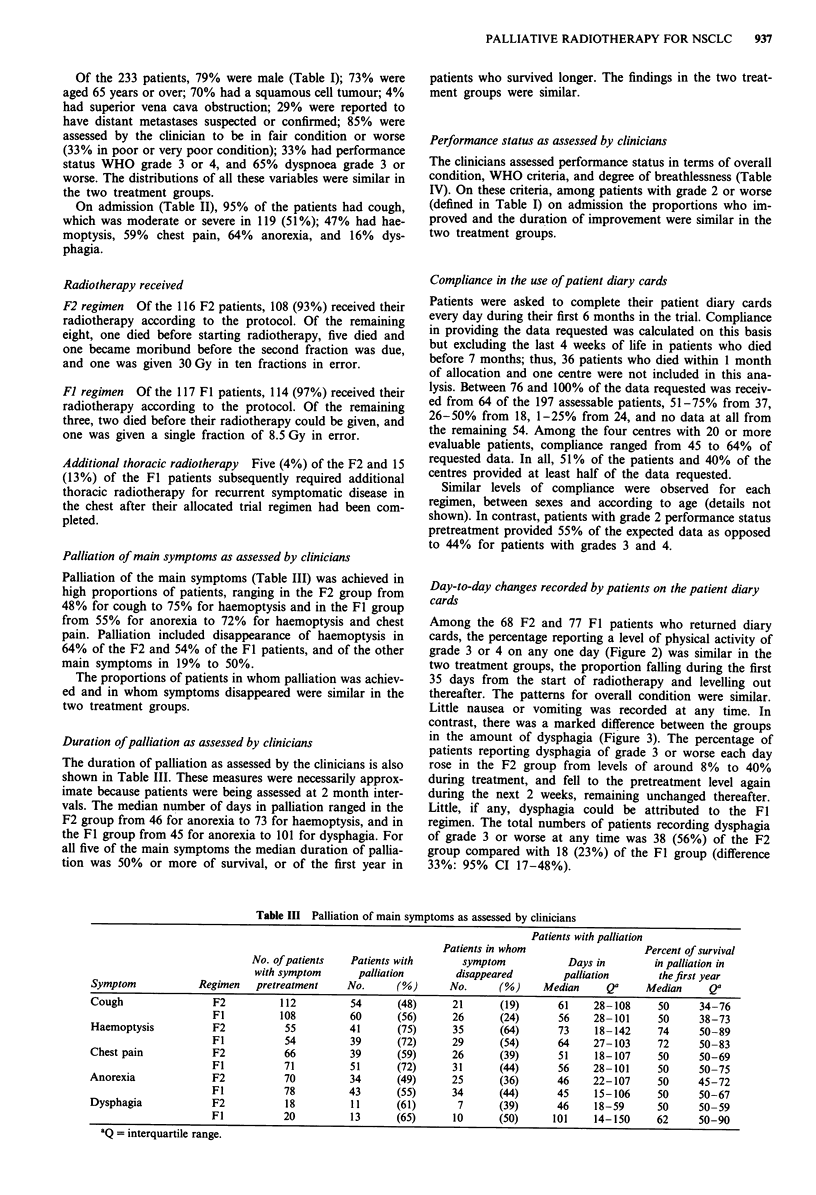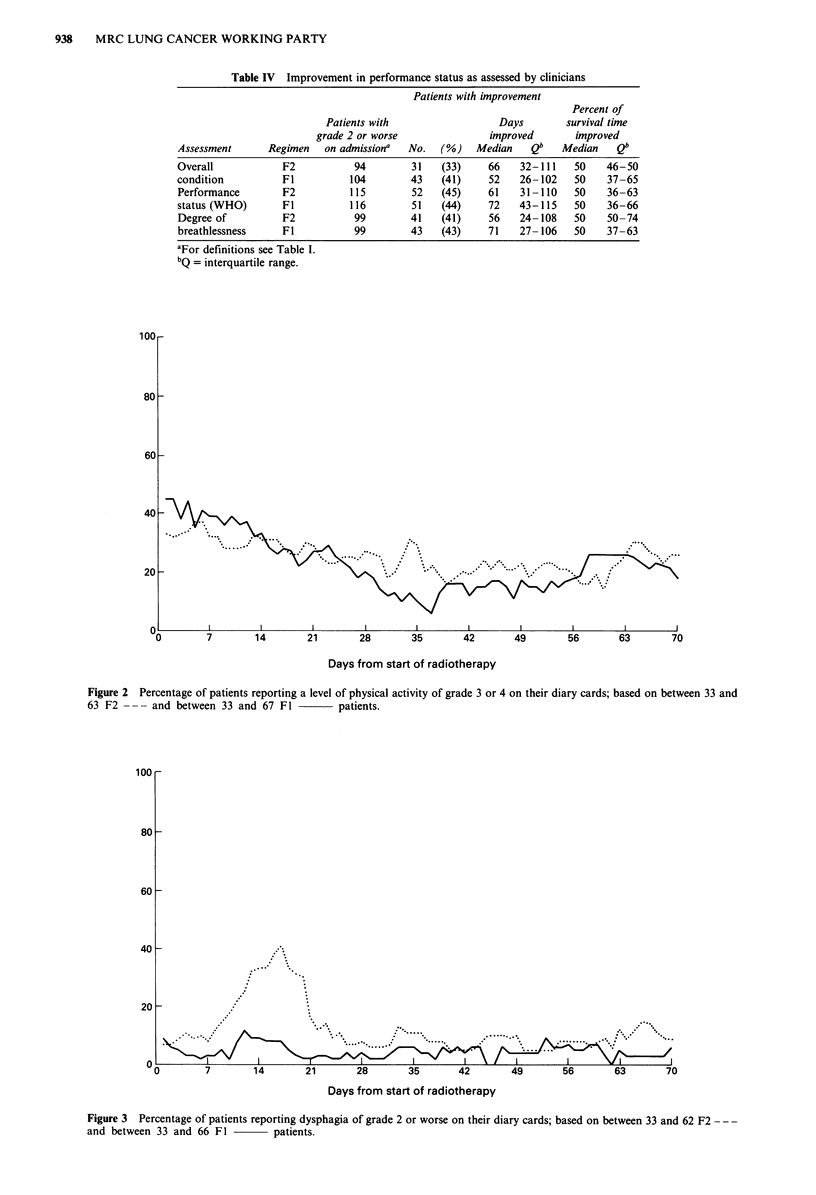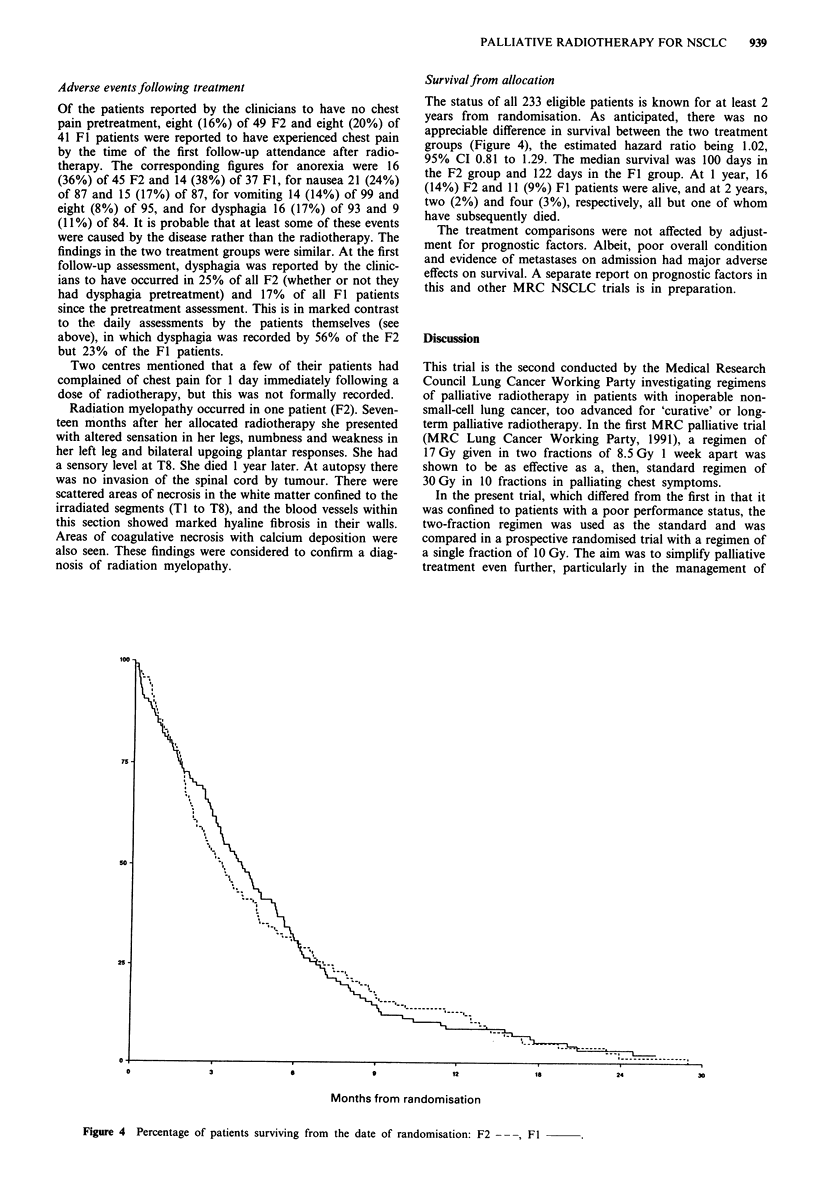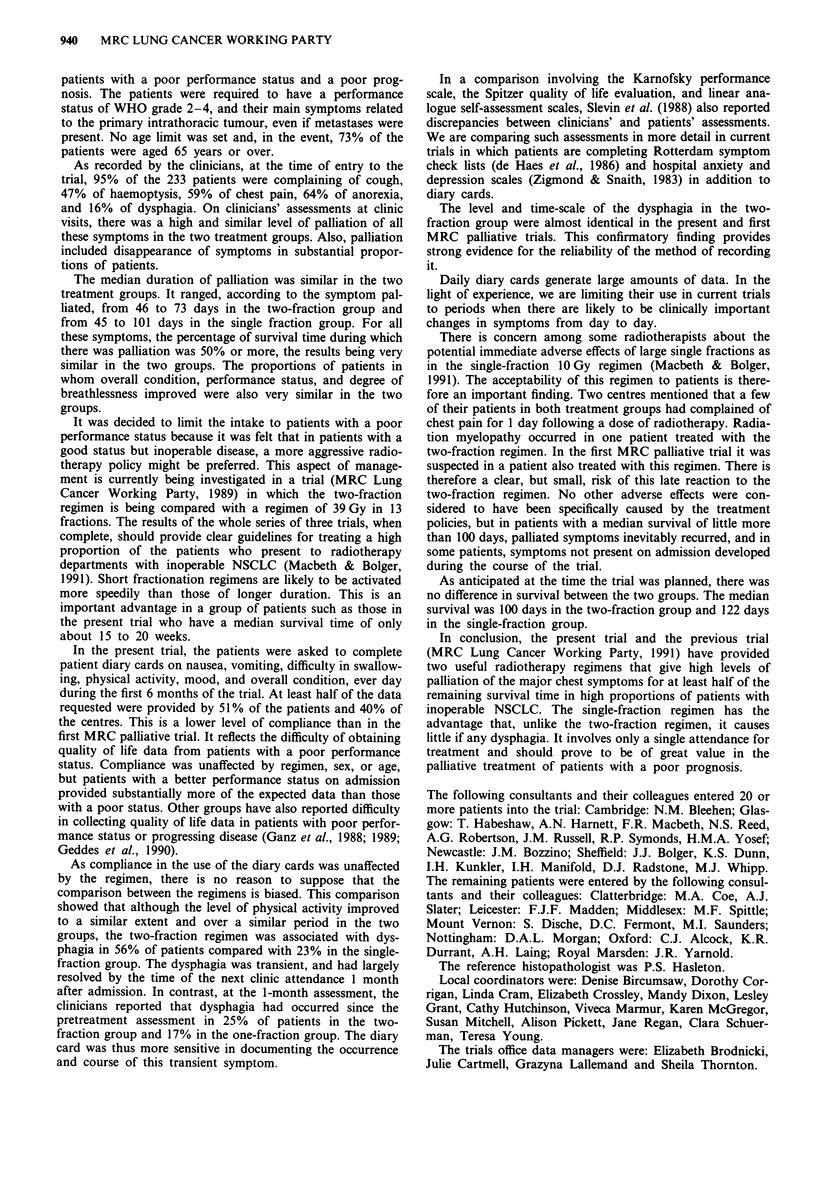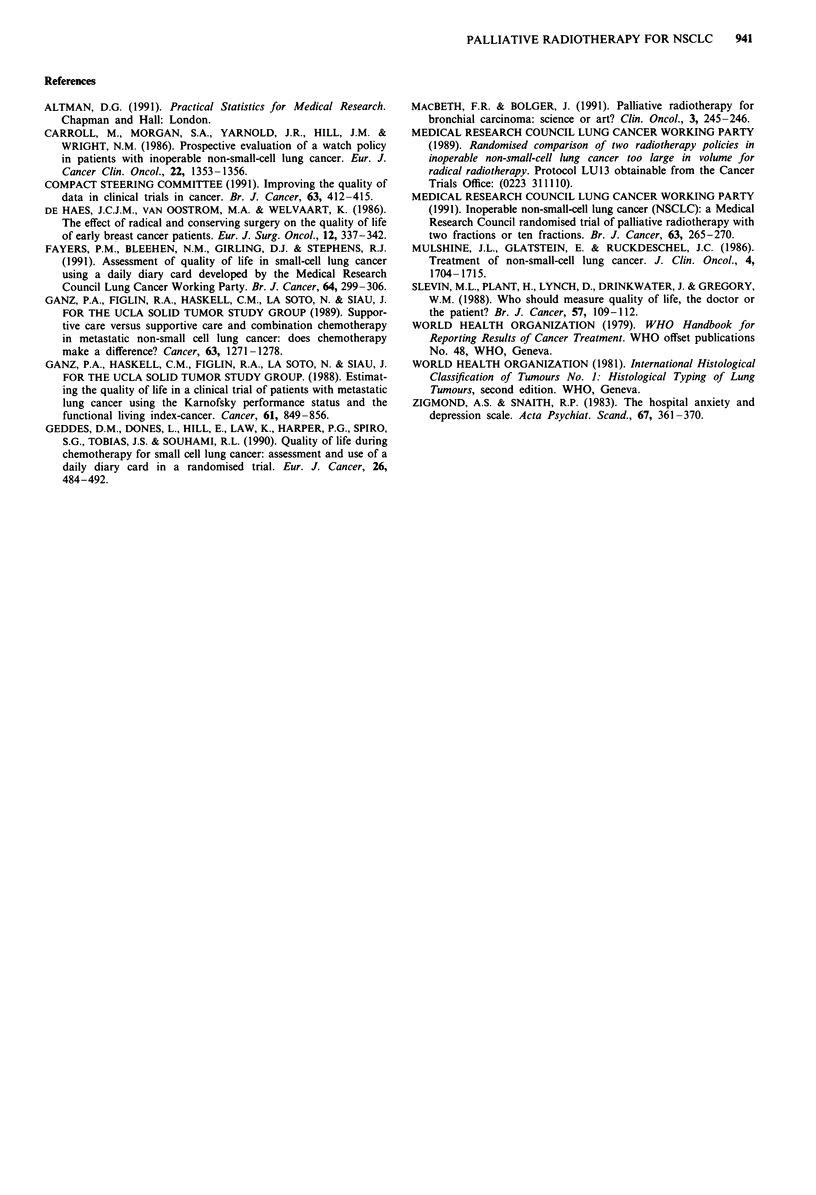# A Medical Research Council (MRC) randomised trial of palliative radiotherapy with two fractions or a single fraction in patients with inoperable non-small-cell lung cancer (NSCLC) and poor performance status. Medical Research Council Lung Cancer Working Party.

**DOI:** 10.1038/bjc.1992.196

**Published:** 1992-06

**Authors:** 

## Abstract

Two policies of palliative thoracic radiotherapy for NSCLC have been compared in a randomised multicentre controlled trial aimed at simplifying the palliative treatment of patients with poor performance status. A total of 235 patients were entered. They had inoperable, microscopically confirmed disease, too advanced for 'curative' radiotherapy. Their main symptoms were related to the primary intrathoracic tumour even if metastases were present, and they had a poor performance status. Patients were allocated at random to regimens of either 17 Gy given in two fractions of 8.5 Gy 1 week apart (F2 regimen, 117 patients), or a single fraction of 10 Gy (F1 regimen, 118 patients). Two patients (one in each group) were excluded from all analyses because they were found to have had previously treated malignant disease and had been admitted in error. On admission, 95% of the 233 eligible patients had cough, 47% haemoptysis, 59% chest pain, 64% anorexia, and 16% dysphagia. As assessed by the clinicians, these symptoms were palliated in high proportions of patients, ranging in the F2 group from 48% for cough to 75% for haemoptysis, and in the F1 group from 55% for anorexia to 72% for haemoptysis and chest pain. For all five symptoms the median duration of palliation was 50% or more of survival. All these results were similar in the two treatment groups. In contrast, on daily assessment by the patients using a diary card, those treated with the F2 regimen experienced substantially more dysphagia, which was recorded in 56% of the patients compared with 23% in the F1 group (difference 33%: 95% confidence interval 17-48%). The median survival from randomisation was 100 days in the F2 group and 122 days in the F1 group. The F1 regimen, as it requires only a single attendance for treatment, is recommended as a palliative regimen for patients with inoperable NSCLC and a poor performance status.


Br. J. Cancer (1992), 65, 934–941 © Macmillan Press Ltd., 1992

### A Medical Research Council (MRC) randomised trial of palliative radiotherapy with two fractions or a single fraction in patients with inoperable non-small-cell lung cancer (NSCLC) and poor performance status

Medical Research Council Lung Cancer Working Party*
Prepared on behalf of the working party and all its collaborators by: N.M. Bleehen, D.J. Girling, D. Machin and R.J. Stephens

**Summary** Two policies of palliative thoracic radiotherapy for NSCLC have been compared in a randomised multicentre controlled trial aimed at simplifying the palliative treatment of patients with poor performance status. A total of 235 patients were entered. They had inoperable, microscopically confirmed disease, too advanced for 'curative' radiotherapy. Their main symptoms were related to the primary intrathoracic tumour even if metastases were present, and they had a poor performance status. Patients were allocated at random to regimens of either 17 Gy given in two fractions of 8.5 Gy 1 week apart (F2 regimen, 117 patients), or a single fraction of 10 Gy (F1 regimen, 118 patients). Two patients (one in each group) were excluded from all analyses because they were found to have had previously treated malignant disease and had been admitted in error. On admission, 95% of the 233 eligible patients had cough, 47% haemoptysis, 59% chest pain, 64% anorexia, and 16% dysphagia. As assessed by the clinicians, these symptoms were palliated in high proportions of patients, ranging in the F2 group from 48% for cough to 75% for haemoptysis, and in the F1 group from 55% for anorexia to 72% for haemoptysis and chest pain. For all five symptoms the median duration of palliation was 50% or more of survival. All these results were similar in the two treatment groups. In contrast, on daily assessment by the patients using a diary card, those treated with the F2 regimen experienced substantially more dysphagia, which was recorded in 56% of the patients compared with 23% in the F1 group (difference 33%: 95% confidence interval 17–48%). The median survival from randomisation was 100 days in the F2 group and 122 days in the F1 group. The F1 regimen, as it requires only a single attendance for treatment, is recommended as a palliative regimen for patients with inoperable NSCLC and a poor performance status.

The majority of patients with inoperable NSCLC have tumour already too advanced for radical radiotherapy at the time they present, but require palliative treatment for major symptoms related to intrathoracic tumour (Carrol *et al.*, 1986). It is usual to treat such patients, either at first presentation or when significant symptoms develop, with a course of palliative radiotherapy (Mulshine *et al.*, 1986).

An earlier randomised clinical trial (MRC Lung Cancer Working Party, 1991) showed that in the management of patients with previously untreated, inoperable NSCLC too advanced for 'curative' or long-term palliative radiotherapy, effective palliation of chest symptoms was achieved with only two fractions of 8.5 Gy given 1 week apart (total dose 17 Gy). This regimen was as effective as a regimen considered to be conventional treatment at the time, namely a multifractionated regimen of 30 Gy in ten equal fractions (or the biologically equivalent dose of 27 Gy in six equal fractions) given daily except at weekends. The main symptoms, namely cough, haemoptysis, chest pain, and anorexia, were palliated in high and similar proportions of patients in the two treatment groups, and the main adverse effect of the radiotherapy, namely dysphagia, was of similar frequency and severity as recorded daily by the patients themselves using a diary card. Performance status on admission had a major prognostic effect, but there was no difference in survival between the two randomised groups. The two-fraction regimen was therefore recommended as it required patients to attend only twice for treatment, and greatly reduced the cost of treatment in terms of machine-time and staff.

The aim of the present study was to investigate whether only a single fraction of radiotherapy could provide equally good palliation as the two-fraction regimen used in the previous study, in the management of patients with inoperable NSCLC, a poor performance status, and whose main symptoms were related to intrathoracic tumour. A survival difference was not anticipated. In a study of purely palliative radiotherapy, the intake was restricted to patients with a poor performance status, because performance status on admission was known to affect the duration of survival. In patients with a better status a higher-dose regimen aimed not only at palliation but also at prolonging survival might be preferred.

### Methods

### *Eligibility*

Patients of either sex and any age were eligible for the trial if they had previously untreated, inoperable, histologically or cytologically proved lung carcinoma of any histological type except small-cell. It was required that their disease was considered too advanced for 'curative' or long-term palliative radiotherapy, but that survival was anticipated to exceed 2 months from admission. They had to have poor performance status, namely WHO grade 2–4 (World Health Organization, 1979), and their main symptoms related to the primary intrathoracic tumour, even if metastases were present. Local ethics committee approval of the protocol and individual patient consent were required.

The diagnoses were made by the histopathologists from the referring centres according to the WHO classification (World Health Organization, 1981). To obtain uniformity of classification, the slides were later examined by a single reference histopathologist.

*Members: N.M. Bleehen (Chairman until October 1989), J.J. Bolger, D.J. Girling (Secretary), P.S. Hasleton, P. Hopwood, F.R. Macbeth, D. Machin (Statistician), K. Moghissi, M. Saunders, R.J. Stephens, N. Thatcher (Chairman from October 1989), R.J. White.
Correspondence: D.J. Girling, MRC Cancer Trials Office, 1 Brooklands Avenue, Cambridge CB2 2BB, UK.
Received 22 October 1991; and in revised form 13 February 1992.

*Pretreatment investigations*

The pretreatment investigations included clinical examination, a postero-anterior chest radiograph, and measurement of the blood haemoglobin concentration, total white cell and platelet counts.

*Treatment allocation*

Clinicians telephoned the MRC Cancer Trials Office and patients were randomly allocated to one or other of two treatment regimens using a minimisation procedure, stratifying for histological type and admitting radiotherapist.

*Two-fraction regimen: F2* The patients allocated to the F2 regimen were given megavoltage radiotherapy to a total midline dose of 17 Gy, calculated without air correction, in two fractions of 8.5 Gy 1 week apart.

*One-fraction regimen: F1* The patients allocated to the F1 regimen were given megavoltage radiotherapy in a single midline dose of 10 Gy, calculated without air correction.

In both F2 and F1 groups, the radiotherapy was delivered through opposing portals to the primary site and mediastinal lymph nodes. The field included the loco-regional tumour volume with a margin of not more than 1 cm, the area, allowing for leading, not exceeding 200 $cm^2$.

Concurrent steroid administration was recommended for patients with superior vena cava obstruction, and was also permitted in other patients.

*Reports and investigations*

A progress report on each patient was completed 1 month and 2 months after the date of start of radiotherapy, then once every 2 months up to 1 year, and thereafter once every 6 months. These reports included details of the treatment given, and details of any metastases.

*Assessment of palliation by clinicians*

The clinician's assessments of the patient's overall condition, performance status, and degree of breathlessness were recorded at each attendance according to the categories shown in Table I. The clinician also asked the patient about the occurrence and severity, since the previous attendance, of the symptoms listed in Table II, and also of nausea, vomiting, and other symptoms, recording the answers as none, mild, moderate, or severe.

*Daily assessment by patients*

For their first 6 months in the study, the patients completed an MRC patient diary card (Fayers *et al.*, 1991) every evening after their last meal, recording how they had been feeling during the previous 24 h using a numerical code (Figure 1).

*Statistical methods*

Palliation of a symptom was defined as disappearance of the symptom or improvement by one or more categories (from

**Table I** General characteristics of the 233 eligible patients on admission

| Characteristic | F2 No. | F2 (%) | F1 No. | F1 (%) | Total No. | Total (%) |
|---|---|---|---|---|---|---|
| Sex: Male | 93 | (80) | 92 | (79) | 185 | (79) |
| Age (years): | | | | | | |
| –45 | 0 | (0) | 1 | (1) | 1 | (0) |
| 45–54 | 3 | (3) | 6 | (5) | 9 | (4) |
| 55–64 | 26 | (22) | 28 | (24) | 54 | (23) |
| 65–74 | 60 | (52) | 56 | (48) | 116 | (50) |
| 75 + | 27 | (23) | 26 | (22) | 53 | (23) |
| Histology (assessed locally) | | | | | | |
| Squamous | 82 | (71) | 81 | (69) | 163 | (70) |
| Adenocarcinoma | 10 | (9) | 11 | (9) | 21 | (9) |
| Large-cell | 13 | (11) | 14 | (12) | 27 | (12) |
| Other | 3 | (3) | 3 | (3) | 6 | (3) |
| Untyped | 8 | (7) | 8 | (7) | 16 | (7) |
| Superior vena cava obstruction | | | | | | |
| Present | 5 | (4) | 4 | (4) | 9 | (4) |
| Not known | 2 | | 3 | | 5 | |
| Distant metastases | | | | | | |
| Present | 30 | (26) | 38 | (33) | 68 | (29) |
| Not known | 0 | | 1 | | 1 | |
| Overall condition | | | | | | |
| 0. Excellent | 1 | (1) | 1 | (1) | 2 | (1) |
| 1. Good | 20 | (17) | 12 | (10) | 32 | (14) |
| 2. Fair | 55 | (48) | 67 | (57) | 122 | (53) |
| 3. Poor | 37 | (32) | 32 | (27) | 69 | (30) |
| 4. Very poor | 2 | (2) | 5 | (4) | 7 | (3) |
| Not known | 1 | | 0 | | 1 | |
| Performance status (WHO, 1979): | | | | | | |
| 2. up and about >50% of waking hours, unable to work, capable of all self-care | 76 | (66) | 78 | (67) | 154 | (67) |
| 3. confined to bed or chair >50% of waking hours, limited self-care | 34 | (30) | 32 | (28) | 66 | (29) |
| 4. confined to bed or chair, no self-care | 5 | (4) | 6 | (5) | 11 | (5) |
| Not known | 1 | | 1 | | 2 | |
| Degree of breathlessness | | | | | | |
| 0. Climbs hills or stairs without dyspnoea | 2 | (2) | 4 | (3) | 6 | (3) |
| 1. Walks any distance on flat without dyspnoea | 15 | (13) | 13 | (11) | 28 | (12) |
| 2. Walks over 100 yards without dyspnoea | 23 | (20) | 24 | (21) | 47 | (20) |
| 3. Dyspnoea on walking 100 yards or less | 48 | (41) | 43 | (37) | 91 | (39) |
| 4. Dyspnoea on mild exertion, e.g. undressing | 28 | (24) | 32 | (28) | 60 | (26) |
| Not known | 0 | | 1 | | 1 | |

**Table II** Main symptoms on admission as recorded by the clinicians

| *Symptom* | *F2* No. | *F2* (%) | *F1* No. | *F1* (%) | *Total* No. | *Total* (%) |
|---|---|---|---|---|---|---|
| Cough[a] | | | | | | |
| None | 3 | (3) | 9 | (8) | 12 | (5) |
| Mild | 51 | (44) | 50 | (43) | 101 | (44) |
| Moderate | 54 | (47) | 50 | (43) | 104 | (45) |
| Severe | 7 | (6) | 8 | (7) | 15 | (6) |
| Haemoptysis | | | | | | |
| None | 61 | (53) | 63 | (54) | 124 | (53) |
| Mild | 35 | (30) | 29 | (25) | 64 | (27) |
| Moderate | 19 | (16) | 24 | (21) | 43 | (18) |
| Severe | 1 | (1) | 1 | (1) | 2 | (1) |
| Chest pain | | | | | | |
| None | 50 | (43) | 46 | (39) | 96 | (41) |
| Mild | 30 | (26) | 33 | (28) | 63 | (27) |
| Moderate | 30 | (26) | 31 | (26) | 61 | (26) |
| Severe | 6 | (5) | 7 | (6) | 13 | (6) |
| Anorexia[a] | | | | | | |
| None | 45 | (39) | 39 | (33) | 84 | (36) |
| Mild | 32 | (28) | 42 | (36) | 74 | (32) |
| Moderate | 31 | (27) | 34 | (29) | 65 | (28) |
| Severe | 7 | (6) | 2 | (2) | 9 | (4) |
| Dysphagia[a] | | | | | | |
| None | 97 | (84) | 97 | (83) | 194 | (84) |
| Mild | 11 | (10) | 11 | (9) | 22 | (9) |
| Moderate | 3 | (3) | 5 | (4) | 8 | (3) |
| Severe | 4 | (3) | 4 | (3) | 8 | (3) |

[a]1 (F2) not known.

mild to none, from moderate to mild or none, or from severe to moderate, mild, or none), at one or more assessment. Duration of palliation is expressed (i) as the median duration of palliation and (ii) as the percentage of patient survival time during which there was palliation. The variation in these two statistics is expressed by the interquartile range (Q), which is the range of the two middle quarters of the results. Ninety-five per cent confidence intervals (CI) for proportions are given where appropriate. The Kaplan-Meier estimate was used to calculate survival curves and a log-rank procedure was used to calculate confidence intervals for the observed differences. The effect of factors for prognosis on survival was assessed by a proportional hazards regression model as described by Altman (1991). The trial data were managed using the COMPACT program (COMPACT Steering Committee, 1991).

### Results

### *Patients in the study*

Between February 1988 and September 1989, 235 patients were randomised from 11 centres in the United Kingdom. Two patients (1 F2, 1 F1) were found to have had previously treated malignant disease and were therefore not eligible for the trial. There remain 233 (116 F2, 117 F1) for analysis on an intention to treat basis. All had NSCLC diagnosed locally, but in six (3 F2, 3 F1) the subsequent assessment by the reference histopathologist was small-cell lung cancer.

N.B. Please fill in date of first entry on this card:

Date 1.3.88

WEEK 1

| | Mon | Tue | Wed | Thu | Fri | Sat | Sun |
|---|---|---|---|---|---|---|---|
| DATE | | 1 | 2 | 3 | 4 | 5 | 6 |
| NAUSEA | | 1 | 1 | 1 | 1 | 1 | 1 |
| VOMITING | | 1 | 1 | 1 | 1 | 1 | 1 |
| DIFFICULTY IN SWALLOWING | | 2 | 3 | 4 | 3 | 3 | 1 |
| ACTIVITY | | 4 | 5 | 5 | 4 | 4 | 4 |
| MOOD | | 4 | 4 | 3 | 3 | 3 | 2 |
| OVERALL CONDITION | | 4 | 4 | 3 | 3 | 3 | 3 |
| Tick if treatment given in hospital | | ✓ | ✓ | ✓ | ✓ | ✓ | ✓ |

Please give details of any other problems or changes in your general health:–

BREATHING BAD.
OFF FOOD.

CODING

NAUSEA:

1. None

2. Mild

3. Moderate

4. Severe

VOMITING:
1. None
2. Sick once
3. Sick 2 or 3 times
4. Sick 4 or more times

DIFFICULTY IN SWALLOWING:
1. None
2. Mild soreness only
3. Can swallow solids with difficulty
4. Cannot swallow solids
5. Cannot swallow liquids

ACTIVITY:
1. Normal work/housework
2. Normal work but with effort
3. Reduced activity but not confined to home
4. Confined to home or hospital
5. Confined to bed

MOOD:
1. Very happy
2. Happy
3. Average
4. Miserable
5. Very miserable

OVERALL CONDITION:
1. Very well
2. Well
3. Fair
4. Poor
5. Very ill

**Figure 1** An example of 1 week from a patient diary card. Each card covered 5 weeks.

Of the 233 patients, 79% were male (Table I); 73% were aged 65 years or over; 70% had a squamous cell tumour; 4% had superior vena cava obstruction; 29% were reported to have distant metastases suspected or confirmed; 85% were assessed by the clinician to be in fair condition or worse (33% in poor or very poor condition); 33% had performance status WHO grade 3 or 4, and 65% dyspnoea grade 3 or worse. The distributions of all these variables were similar in the two treatment groups.

On admission (Table II), 95% of the patients had cough, which was moderate or severe in 119 (51%); 47% had haemoptysis, 59% chest pain, 64% anorexia, and 16% dysphagia.

### *Radiotherapy received*

*F2 regimen* Of the 116 F2 patients, 108 (93%) received their radiotherapy according to the protocol. Of the remaining eight, one died before starting radiotherapy, five died and one became moribund before the second fraction was due, and one was given 30 Gy in ten fractions in error.

*F1 regimen* Of the 117 F1 patients, 114 (97%) received their radiotherapy according to the protocol. Of the remaining three, two died before their radiotherapy could be given, and one was given a single fraction of 8.5 Gy in error.

*Additional thoracic radiotherapy* Five (4%) of the F2 and 15 (13%) of the F1 patients subsequently required additional thoracic radiotherapy for recurrent symptomatic disease in the chest after their allocated trial regimen had been completed.

### *Palliation of main symptoms as assessed by clinicians*

Palliation of the main symptoms (Table III) was achieved in high proportions of patients, ranging in the F2 group from 48% for cough to 75% for haemoptysis and in the F1 group from 55% for anorexia to 72% for haemoptysis and chest pain. Palliation included disappearance of haemoptysis in 64% of the F2 and 54% of the F1 patients, and of the other main symptoms in 19% to 50%.

The proportions of patients in whom palliation was achieved and in whom symptoms disappeared were similar in the two treatment groups.

### *Duration of palliation as assessed by clinicians*

The duration of palliation as assessed by the clinicians is also shown in Table III. These measures were necessarily approximate because patients were being assessed at 2 month intervals. The median number of days in palliation ranged in the F2 group from 46 for anorexia to 73 for haemoptysis, and in the F1 group from 45 for anorexia to 101 for dysphagia. For all five of the main symptoms the median duration of palliation was 50% or more of survival, or of the first year in patients who survived longer. The findings in the two treatment groups were similar.

### *Performance status as assessed by clinicians*

The clinicians assessed performance status in terms of overall condition, WHO criteria, and degree of breathlessness (Table IV). On these criteria, among patients with grade 2 or worse (defined in Table I) on admission the proportions who improved and the duration of improvement were similar in the two treatment groups.

### *Compliance in the use of patient diary cards*

Patients were asked to complete their patient diary cards every day during their first 6 months in the trial. Compliance in providing the data requested was calculated on this basis but excluding the last 4 weeks of life in patients who died before 7 months; thus, 36 patients who died within 1 month of allocation and one centre were not included in this analysis. Between 76 and 100% of the data requested was received from 64 of the 197 assessable patients, 51–75% from 37, 26–50% from 18, 1–25% from 24, and no data at all from the remaining 54. Among the four centres with 20 or more evaluable patients, compliance ranged from 45 to 64% of requested data. In all, 51% of the patients and 40% of the centres provided at least half of the data requested.

Similar levels of compliance were observed for each regimen, between sexes and according to age (details not shown). In contrast, patients with grade 2 performance status pretreatment provided 55% of the expected data as opposed to 44% for patients with grades 3 and 4.

### *Day-to-day changes recorded by patients on the patient diary cards*

Among the 68 F2 and 77 F1 patients who returned diary cards, the percentage reporting a level of physical activity of grade 3 or 4 on any one day (Figure 2) was similar in the two treatment groups, the proportion falling during the first 35 days from the start of radiotherapy and levelling out thereafter. The patterns for overall condition were similar. Little nausea or vomiting was recorded at any time. In contrast, there was a marked difference between the groups in the amount of dysphagia (Figure 3). The percentage of patients reporting dysphagia of grade 3 or worse each day rose in the F2 group from levels of around 8% to 40% during treatment, and fell to the pretreatment level again during the next 2 weeks, remaining unchanged thereafter. Little, if any, dysphagia could be attributed to the F1 regimen. The total numbers of patients recording dysphagia of grade 3 or worse at any time was 38 (56%) of the F2 group compared with 18 (23%) of the F1 group (difference 33%: 95% CI 17–48%).

**Table III** Palliation of main symptoms as assessed by clinicians

| | | | Patients with palliation | | | | | | | |
|---|---|---|---|---|---|---|---|---|---|---|
| | | | | | | | | | | |
| *Symptom* | *Regimen* | *No. of patients with symptom pretreatment* | *Patients with palliation No.* | *(%)* | *Patients in whom symptom disappeared No.* | *(%)* | *Days in palliation Median* | *Q*[a] | *Percent of survival in palliation in the first year Median* | *Q*[a] |
| Cough | F2 | 112 | 54 | (48) | 21 | (19) | 61 | 28–108 | 50 | 34–76 |
| | F1 | 108 | 60 | (56) | 26 | (24) | 56 | 28–101 | 50 | 38–73 |
| Haemoptysis | F2 | 55 | 41 | (75) | 35 | (64) | 73 | 18–142 | 74 | 50–89 |
| | F1 | 54 | 39 | (72) | 29 | (54) | 64 | 27–103 | 72 | 50–83 |
| Chest pain | F2 | 66 | 39 | (59) | 26 | (39) | 51 | 18–107 | 50 | 50–69 |
| | F1 | 71 | 51 | (72) | 31 | (44) | 56 | 28–101 | 50 | 50–75 |
| Anorexia | F2 | 70 | 34 | (49) | 25 | (36) | 46 | 22–107 | 50 | 45–72 |
| | F1 | 78 | 43 | (55) | 34 | (44) | 45 | 15–106 | 50 | 50–67 |
| Dysphagia | F2 | 18 | 11 | (61) | 7 | (39) | 46 | 18–59 | 50 | 50–59 |
| | F1 | 20 | 13 | (65) | 10 | (50) | 101 | 14–150 | 62 | 50–90 |

[a]Q = interquartile range.

**Table IV** Improvement in performance status as assessed by clinicians

| Assessment | Regimen | Patients with grade 2 or worse on admission[a] | Patients with improvement No. | (%) | Days improved Median | $Q^b$ | Percent of survival time improved Median | $Q^b$ |
|---|---|---|---|---|---|---|---|---|
| Overall | F2 | 94 | 31 | (33) | 66 | 32-111 | 50 | 46-50 |
| condition | F1 | 104 | 43 | (41) | 52 | 26-102 | 50 | 37-65 |
| Performance | F2 | 115 | 52 | (45) | 61 | 31-110 | 50 | 36-63 |
| status (WHO) | F1 | 116 | 51 | (44) | 72 | 43-115 | 50 | 36-66 |
| Degree of | F2 | 99 | 41 | (41) | 56 | 24-108 | 50 | 50-74 |
| breathlessness | F1 | 99 | 43 | (43) | 71 | 27-106 | 50 | 37-63 |

[a]For definitions see Table I.
[b]Q = interquartile range.

**Figure 2** Percentage of patients reporting a level of physical activity of grade 3 or 4 on their diary cards; based on between 33 and 63 F2 --- and between 33 and 67 F1 ——— patients.

**Figure 3** Percentage of patients reporting dysphagia of grade 2 or worse on their diary cards; based on between 33 and 62 F2 --- and between 33 and 66 F1 ——— patients.

*Adverse events following treatment*

Of the patients reported by the clinicians to have no chest pain pretreatment, eight (16%) of 49 F2 and eight (20%) of 41 F1 patients were reported to have experienced chest pain by the time of the first follow-up attendance after radiotherapy. The corresponding figures for anorexia were 16 (36%) of 45 F2 and 14 (38%) of 37 F1, for nausea 21 (24%) of 87 and 15 (17%) of 87, for vomiting 14 (14%) of 99 and eight (8%) of 95, and for dysphagia 16 (17%) of 93 and 9 (11%) of 84. It is probable that at least some of these events were caused by the disease rather than the radiotherapy. The findings in the two treatment groups were similar. At the first follow-up assessment, dysphagia was reported by the clinicians to have occurred in 25% of all F2 (whether or not they had dysphagia pretreatment) and 17% of all F1 patients since the pretreatment assessment. This is in marked contrast to the daily assessments by the patients themselves (see above), in which dysphagia was recorded by 56% of the F2 but 23% of the F1 patients.

Two centres mentioned that a few of their patients had complained of chest pain for 1 day immediately following a dose of radiotherapy, but this was not formally recorded.

Radiation myelopathy occurred in one patient (F2). Seventeen months after her allocated radiotherapy she presented with altered sensation in her legs, numbness and weakness in her left leg and bilateral upgoing plantar responses. She had a sensory level at T8. She died 1 year later. At autopsy there was no invasion of the spinal cord by tumour. There were scattered areas of necrosis in the white matter confined to the irradiated segments (T1 to T8), and the blood vessels within this section showed marked hyaline fibrosis in their walls. Areas of coagulative necrosis with calcium deposition were also seen. These findings were considered to confirm a diagnosis of radiation myelopathy.

*Survival from allocation*

The status of all 233 eligible patients is known for at least 2 years from randomisation. As anticipated, there was no appreciable difference in survival between the two treatment groups (Figure 4), the estimated hazard ratio being 1.02, 95% CI 0.81 to 1.29. The median survival was 100 days in the F2 group and 122 days in the F1 group. At 1 year, 16 (14%) F2 and 11 (9%) F1 patients were alive, and at 2 years, two (2%) and four (3%), respectively, all but one of whom have subsequently died.

The treatment comparisons were not affected by adjustment for prognostic factors. Albeit, poor overall condition and evidence of metastases on admission had major adverse effects on survival. A separate report on prognostic factors in this and other MRC NSCLC trials is in preparation.

### Discussion

This trial is the second conducted by the Medical Research Council Lung Cancer Working Party investigating regimens of palliative radiotherapy in patients with inoperable non-small-cell lung cancer, too advanced for 'curative' or long-term palliative radiotherapy. In the first MRC palliative trial (MRC Lung Cancer Working Party, 1991), a regimen of 17 Gy given in two fractions of 8.5 Gy 1 week apart was shown to be as effective as a, then, standard regimen of 30 Gy in 10 fractions in palliating chest symptoms.

In the present trial, which differed from the first in that it was confined to patients with a poor performance status, the two-fraction regimen was used as the standard and was compared in a prospective randomised trial with a regimen of a single fraction of 10 Gy. The aim was to simplify palliative treatment even further, particularly in the management of

**Figure 4** Percentage of patients surviving from the date of randomisation: F2 – – –, F1 ———.

patients with a poor performance status and a poor prognosis. The patients were required to have a performance status of WHO grade 2–4, and their main symptoms related to the primary intrathoracic tumour, even if metastases were present. No age limit was set and, in the event, 73% of the patients were aged 65 years or over.

As recorded by the clinicians, at the time of entry to the trial, 95% of the 233 patients were complaining of cough, 47% of haemoptysis, 59% of chest pain, 64% of anorexia, and 16% of dysphagia. On clinicians' assessments at clinic visits, there was a high and similar level of palliation of all these symptoms in the two treatment groups. Also, palliation included disappearance of symptoms in substantial proportions of patients.

The median duration of palliation was similar in the two treatment groups. It ranged, according to the symptom palliated, from 46 to 73 days in the two-fraction group and from 45 to 101 days in the single fraction group. For all these symptoms, the percentage of survival time during which there was palliation was 50% or more, the results being very similar in the two groups. The proportions of patients in whom overall condition, performance status, and degree of breathlessness improved were also very similar in the two groups.

It was decided to limit the intake to patients with a poor performance status because it was felt that in patients with a good status but inoperable disease, a more aggressive radiotherapy policy might be preferred. This aspect of management is currently being investigated in a trial (MRC Lung Cancer Working Party, 1989) in which the two-fraction regimen is being compared with a regimen of 39 Gy in 13 fractions. The results of the whole series of three trials, when complete, should provide clear guidelines for treating a high proportion of the patients who present to radiotherapy departments with inoperable NSCLC (Macbeth & Bolger, 1991). Short fractionation regimens are likely to be activated more speedily than those of longer duration. This is an important advantage in a group of patients such as those in the present trial who have a median survival time of only about 15 to 20 weeks.

In the present trial, the patients were asked to complete patient diary cards on nausea, vomiting, difficulty in swallowing, physical activity, mood, and overall condition, ever day during the first 6 months of the trial. At least half of the data requested were provided by 51% of the patients and 40% of the centres. This is a lower level of compliance than in the first MRC palliative trial. It reflects the difficulty of obtaining quality of life data from patients with a poor performance status. Compliance was unaffected by regimen, sex, or age, but patients with a better performance status on admission provided substantially more of the expected data than those with a poor status. Other groups have also reported difficulty in collecting quality of life data in patients with poor performance status or progressing disease (Ganz *et al.*, 1988; 1989; Geddes *et al.*, 1990).

As compliance in the use of the diary cards was unaffected by the regimen, there is no reason to suppose that the comparison between the regimens is biased. This comparison showed that although the level of physical activity improved to a similar extent and over a similar period in the two groups, the two-fraction regimen was associated with dysphagia in 56% of patients compared with 23% in the single-fraction group. The dysphagia was transient, and had largely resolved by the time of the next clinic attendance 1 month after admission. In contrast, at the 1-month assessment, the clinicians reported that dysphagia had occurred since the pretreatment assessment in 25% of patients in the two-fraction group and 17% in the one-fraction group. The diary card was thus more sensitive in documenting the occurrence and course of this transient symptom.

In a comparison involving the Karnofsky performance scale, the Spitzer quality of life evaluation, and linear analogue self-assessment scales, Slevin *et al.* (1988) also reported discrepancies between clinicians' and patients' assessments. We are comparing such assessments in more detail in current trials in which patients are completing Rotterdam symptom check lists (de Haes *et al.*, 1986) and hospital anxiety and depression scales (Zigmond & Snaith, 1983) in addition to diary cards.

The level and time-scale of the dysphagia in the two-fraction group were almost identical in the present and first MRC palliative trials. This confirmatory finding provides strong evidence for the reliability of the method of recording it.

Daily diary cards generate large amounts of data. In the light of experience, we are limiting their use in current trials to periods when there are likely to be clinically important changes in symptoms from day to day.

There is concern among some radiotherapists about the potential immediate adverse effects of large single fractions as in the single-fraction 10 Gy regimen (Macbeth & Bolger, 1991). The acceptability of this regimen to patients is therefore an important finding. Two centres mentioned that a few of their patients in both treatment groups had complained of chest pain for 1 day following a dose of radiotherapy. Radiation myelopathy occurred in one patient treated with the two-fraction regimen. In the first MRC palliative trial it was suspected in a patient also treated with this regimen. There is therefore a clear, but small, risk of this late reaction to the two-fraction regimen. No other adverse effects were considered to have been specifically caused by the treatment policies, but in patients with a median survival of little more than 100 days, palliated symptoms inevitably recurred, and in some patients, symptoms not present on admission developed during the course of the trial.

As anticipated at the time the trial was planned, there was no difference in survival between the two groups. The median survival was 100 days in the two-fraction group and 122 days in the single-fraction group.

In conclusion, the present trial and the previous trial (MRC Lung Cancer Working Party, 1991) have provided two useful radiotherapy regimens that give high levels of palliation of the major chest symptoms for at least half of the remaining survival time in high proportions of patients with inoperable NSCLC. The single-fraction regimen has the advantage that, unlike the two-fraction regimen, it causes little if any dysphagia. It involves only a single attendance for treatment and should prove to be of great value in the palliative treatment of patients with a poor prognosis.

The following consultants and their colleagues entered 20 or more patients into the trial: Cambridge: N.M. Bleehen; Glasgow: T. Habeshaw, A.N. Harnett, F.R. Macbeth, N.S. Reed, A.G. Robertson, J.M. Russell, R.P. Symonds, H.M.A. Yosef; Newcastle: J.M. Bozzino; Sheffield: J.J. Bolger, K.S. Dunn, I.H. Kunkler, I.H. Manifold, D.J. Radstone, M.J. Whipp. The remaining patients were entered by the following consultants and their colleagues: Clatterbridge: M.A. Coe, A.J. Slater; Leicester: F.J.F. Madden; Middlesex: M.F. Spittle; Mount Vernon: S. Dische, D.C. Fermont, M.I. Saunders; Nottingham: D.A.L. Morgan; Oxford: C.J. Alcock, K.R. Durrant, A.H. Laing; Royal Marsden: J.R. Yarnold.

The reference histopathologist was P.S. Hasleton.

Local coordinators were: Denise Bircumsaw, Dorothy Corrigan, Linda Cram, Elizabeth Crossley, Mandy Dixon, Lesley Grant, Cathy Hutchinson, Viveca Marmur, Karen McGregor, Susan Mitchell, Alison Pickett, Jane Regan, Clara Schuerman, Teresa Young.

The trials office data managers were: Elizabeth Brodnicki, Julie Cartmell, Grazyna Lallemand and Sheila Thornton.

### References

ALTMAN, D.G. (1991). *Practical Statistics for Medical Research*. Chapman and Hall: London.

CARROLL, M., MORGAN, S.A., YARNOLD, J.R., HILL, J.M. & WRIGHT, N.M. (1986). Prospective evaluation of a watch policy in patients with inoperable non-small-cell lung cancer. *Eur. J. Cancer Clin. Oncol.*, **22**, 1353–1356.

COMPACT STEERING COMMITTEE (1991). Improving the quality of data in clinical trials in cancer. *Br. J. Cancer*, **63**, 412–415.

DE HAES, J.C.J.M., VAN OOSTROM, M.A. & WELVAART, K. (1986). The effect of radical and conserving surgery on the quality of life of early breast cancer patients. *Eur. J. Surg. Oncol.*, **12**, 337–342.

FAYERS, P.M., BLEEHEN, N.M., GIRLING, D.J. & STEPHENS, R.J. (1991). Assessment of quality of life in small-cell lung cancer using a daily diary card developed by the Medical Research Council Lung Cancer Working Party. *Br. J. Cancer*, **64**, 299–306.

GANZ, P.A., FIGLIN, R.A., HASKELL, C.M., LA SOTO, N. & SIAU, J. FOR THE UCLA SOLID TUMOR STUDY GROUP (1989). Supportive care versus supportive care and combination chemotherapy in metastatic non-small cell lung cancer: does chemotherapy make a difference? *Cancer*, **63**, 1271–1278.

GANZ, P.A., HASKELL, C.M., FIGLIN, R.A., LA SOTO, N. & SIAU, J. FOR THE UCLA SOLID TUMOR STUDY GROUP. (1988). Estimating the quality of life in a clinical trial of patients with metastatic lung cancer using the Karnofsky performance status and the functional living index-cancer. *Cancer*, **61**, 849–856.

GEDDES, D.M., DONES, L., HILL, E., LAW, K., HARPER, P.G., SPIRO, S.G., TOBIAS, J.S. & SOUHAMI, R.L. (1990). Quality of life during chemotherapy for small cell lung cancer: assessment and use of a daily diary card in a randomised trial. *Eur. J. Cancer*, **26**, 484–492.

MACBETH, F.R. & BOLGER, J. (1991). Palliative radiotherapy for bronchial carcinoma: science or art? *Clin. Oncol.*, **3**, 245–246.

MEDICAL RESEARCH COUNCIL LUNG CANCER WORKING PARTY (1989). *Randomised comparison of two radiotherapy policies in inoperable non-small-cell lung cancer too large in volume for radical radiotherapy*. Protocol LU13 obtainable from the Cancer Trials Office: (0223 311110).

MEDICAL RESEARCH COUNCIL LUNG CANCER WORKING PARTY (1991). Inoperable non-small-cell lung cancer (NSCLC): a Medical Research Council randomised trial of palliative radiotherapy with two fractions or ten fractions. *Br. J. Cancer*, **63**, 265–270.

MULSHINE, J.L., GLATSTEIN, E. & RUCKDESCHEL, J.C. (1986). Treatment of non-small-cell lung cancer. *J. Clin. Oncol.*, **4**, 1704–1715.

SLEVIN, M.L., PLANT, H., LYNCH, D., DRINKWATER, J. & GREGORY, W.M. (1988). Who should measure quality of life, the doctor or the patient? *Br. J. Cancer*, **57**, 109–112.

WORLD HEALTH ORGANIZATION (1979). *WHO Handbook for Reporting Results of Cancer Treatment*. WHO offset publications No. 48, WHO, Geneva.

WORLD HEALTH ORGANIZATION (1981). *International Histological Classification of Tumours No. 1: Histological Typing of Lung Tumours*, second edition. WHO, Geneva.

ZIGMOND, A.S. & SNAITH, R.P. (1983). The hospital anxiety and depression scale. *Acta Psychiat. Scand.*, **67**, 361–370.